# Pharmacological reacidification of lysosomes attenuates intraneuronal amyloidosis, early neuron death, and amyloid plaque formation in 5xFAD mice

**DOI:** 10.1002/alz.095538

**Published:** 2025-01-09

**Authors:** Sandeep Malampati, Philip Stavrides, Eunju Im, Chris N. Goulbourne, Panaiyur S. Mohan, Bhaskar C. Das, Ju‐Hyun LEE, Ralph A. Nixon, Philip Stavrides, Eunju Im, Chris N. Goulbourne, Paniyur S. Mohan, Bhaskar C. Das, Ju‐Hyun Lee, Ralph A. Nixon

**Affiliations:** ^1^ Nathan Kline Institute for Psychiatric Research, Orangeburg, NY USA; ^2^ NYU Langone Medical Center, New York, NY USA; ^3^ Amyloid solution Inc, Seongnam‐si, Gye onggi‐do Korea, Republic of (South); ^4^ Long Island University, 75 DeKalb Ave, Brooklyn, NY USA; ^5^ Icahn School of Medicine at Mount Sinai,, New york, NY USA; ^6^ New York University Langone Health, New York, NY USA

## Abstract

**Background:**

Autophagy‐lysosomal pathway (ALP) dysfunction emerges early in Alzheimer’s disease(AD). In mouse AD models, lysosomal acidification deficits impair ALP in neurons, in some inducing massive autolysosome accumulations, intraneuronal amyloid plaque, and early neuronal death yielding an extracellular amyloid plaque. This distinctive neurodegenerative pattern (PANTHOS) emerges in early‐stage AD and is recapitulated in human late‐onset AD (accompanying poster). Restoring lysosomal acidity via β2‐adrenergic receptor (β2‐AR) activation with Isoproterenol (ISO) reverses ALP dysfunction in PSEN1‐FAD patient fibroblasts.

**Method:**

To reverse autolysosomal acidification deficits in vivo, we engineered 33 derivatives using ISO pharmacophore. Candidate compounds compared to ISO underwent screening in AD model cell lines and PSEN1‐FAD fibroblasts. After toxicity and PK/PD studies *in vivo*, a lead compound achieving an effective 11.63 micromolar brain concentration was administered IP for 2 or 4 months in 2‐month 5xFAD mice expressing a ratiometric autophagy/pH LC3 probe (5xFAD/TRGL) or in 2‐month 5xFAD versus vehicle‐treated 5xFAD/TRGL or 5xFAD controls respectively. Effective ISO sustained release pellets were implanted SC in the matched cohorts for 2 months. Outcomes were assessed using multiplex immunocytochemistry, computer‐aided ALP and pH analysis, EM, and behavioral tests (fear conditioning, novel object recognition).

**Result:**

Incubating AD cell lines with the lead compound, restoring lysosome pH by acting through β_2_‐AR mediated, PKA‐dependent pathway, efficiently rescued autophagy. 5xFAD mice treatment with compound or isoproterenol restored neuronal lysosomal acidification and markedly attenuated ALP deficits. Lysosomal re‐acidification strikingly reduced ALP‐related intracellular amyloid/APP‐βCTF/Aβ (PANTHOS) and death of these neurons. Moreover, extracellular plaque burden reduction (>50%) commensurate with reduced neuronal death frequency. Notably, compound induced pH restoration alleviated memory impairments in 5xFAD mice.

**Conclusion:**

These studies establish that lysosomal acidification deficit in the 5xFAD mouse is upstream of, and the likely basis for ALP failure, neuronal death, and amyloid pathology, consistent with the pathological sequence previously documented in multiple mouse AD models. The mechanism of action of β_2_‐AR activators via correcting PKA‐regulated Cl^−^ and H^+^ flux in vATPase‐deficient lysosomes as established in PSEN1‐FAD fibroblasts, likely accounts for autolysosome pH correction in 5xFAD neurons. These findings demonstrate the potential of lysosomal remediation as a therapeutic approach for AD. Grant support: NIA P01AG017617, R01AG062376.

